# Editorial: Non-apoptotic cell death mechanisms and their therapeutic significance

**DOI:** 10.3389/fcell.2022.990285

**Published:** 2022-10-26

**Authors:** Manu Lopus, Jacinta S. D’Souza, Ritu Aneja

**Affiliations:** ^1^ School of Biological Sciences, UM-DAE Centre for Excellence in Basic Sciences, University of Mumbai, Mumbai, India; ^2^ School of Health Professions, The University of Alabama at Birmingham, Birmingham, AL, United States

**Keywords:** non-apoptotic cell death, pyroptosis, ferroptosis, apoptosis, cancer

The field of cell biology has progressed rapidly over the past few decades. From the basic understanding of apoptosis and necrosis in the early 90s, we now have a clearer picture of the different apoptotic and non-apoptotic mechanisms of cell death. With the emergence of advanced techniques and technologies, identifying unconventional cell death mechanisms has become easier in recent years.

Apoptosis is a well-characterized cell death mechanism that helps shape the developing organism, fights infections, optimizes body composition, and prevents hyperproliferative diseases. Over the years, laboratories have noticed many alternative modes of cell death, some of which share features of apoptosis and others bearing little or no resemblance to it (Nirmala and Lopus, 2020). The ability of cancer cells to resist innately programmed or drug-induced apoptosis necessitates identification and targeting of non-apoptotic cell death for effective cancer treatment. Besides apoptosis, there exist several cell death mechanisms. Many of these mechanisms operate to maintain the homeostatic wellbeing of the host organism, while some can as well be detrimental to the host. Some of the well-studied cell death mechanisms include the Ras-mediated, caspase-independent *methuosis,* characterized by the presence of single-membrane-bound large vacuoles; pathogen- and drug-induced *necroptosis* having features of both apoptosis and necrosis; *Netosis*, the classic strategy of neutrophils to trap pathogens by casting some of their content as a net; *pyronecrosis*, which is stimulated by pathogens such as Shigella, and involves inflammasome formation; the autophagy-associated *autosis;* the cannibalistic *entosis,* where a glucose-starved cell engulfs another cell, and the PARP-1-hyperactivation-mediated *parthanatos* (Nirmala and Lopus, 2020). This thematic issue focuses on two distinct cell death mechanisms with considerable therapeutic significance, namely, *pyroptosis* and *ferroptosis*.

Pyroptosis is a non-apoptotic cell death mechanism that manifests as gasdermin-mediated regulated necrosis, characterized by extensive DNA damage and chromatin condensation. Bubbling of the cell membrane followed by flattening due to the oozing of some of the cytoplasmic contents is also observed. Ferroptosis is another non-apoptotic cell death mechanism that is induced by the failure of glutathione-dependent antioxidant regulation. Ferroptosis relies on iron and is characterized by widespread lipid peroxidation in cells. Ferroptosis and pyroptosis play pivotal roles in processes involved in cancer development, prevention, and clinical management. This Research Topic collated a few major studies on pyroptosis and ferroptosis.

Wang et al. used pyroptosis-induced long non-coding RNAs (lncRNAs) to stratify patients with hepatocellular carcinoma (HCC) into high- and low-risk groups. A high-risk score based on the pyroptosis-related lncRNA signature was significantly associated with poor patient survival, after accounting for age and clinical stage. The lncRNA risk score was also associated with overall tumor pathology, with tumors from high- and low-risk patients showing distinct cell infiltrates in the tumor microenvironment. The study provides a novel way of determining a risk score based on a pyroptosis-related lncRNA signature to predict prognosis and response to immunotherapy in patients with HCC. In a similar study, Cao et al. conducted comprehensive genomic and transcriptomic analyses using data to identify mutations in genes and lncRNAs that regulate pyroptosis in ovarian cancer (OC). These analyses revealed that a ‘risk signature’ composed of mutations in pyroptosis-related genes and lncRNAs was associated with prognosis. The signature showed excellent performance in predicting survival based on risk stratification of patients and could be used to guide treatment.

The prognostic value of pyroptosis has also been demonstrated in acute myeloid leukemia (AML). Pan and co-authors (Pan et al.) investigated the genetic and transcriptional alterations in pyroptosis-associated gene signatures in patients with AML and developed a nomogram scoring system based on these signatures. Validation of the scoring system showed that pyroptosis-associated gene signatures could accurately predict the 1-, 3-, and 5-year survival rates of patients with AML. Patients with low expression levels of pyroptosis-associated genes had better survival than those with high levels of pyroptosis gene signatures.

Ferroptosis-related gene signatures have also shown promising prognostic value. In an analysis of data from The Cancer Genome Atlas (TCGA) and RNA sequencing data of 291 ferroptosis-related genes, Song et al. constructed a novel ferroptosis-related gene signature to stratify patients with lung adenocarcinoma (LUAD) into three clusters. Notably, significant survival differences were observed among the three patient subgroups, suggesting that this signature could be used to predict survival in patients with LUAD. In another study on lung cancer, Li et al. studied the mechanisms regulating the induction of ferroptosis. Using data from TCGA and Gene Expression Synthesis (GEO) databases, the authors built a model of ferroptosis-related differentially expressed genes, which they used to stratify patients with non-small cell lung cancer (NSCLC) into different risk groups. Patients in the low-risk group showed a better prognosis than those in the high-risk group, further supporting the prognostic role of ferroptosis-related pathways. Furthermore, they found that AURKA, one of the ferroptosis-related genes, was upregulated in tumors and negatively regulated ferroptosis in NSCLC cells. Treatment with ophiopogonin-B, a traditional Chinese medicine used to treat cancer, led to the upregulation of AURKA and ferroptosis-related markers and downregulation of PHKG2 and SLC7A5 in NSCLC cells. The ability of ophiopogonin-B to induce ferroptosis was partially reversed by AURKA overexpression.

As narrated by Deng et al. in a review article titled ‘Targeting Cancer Cell Ferroptosis to Reverse Immune Checkpoint Inhibitor Therapy Resistance’, ferroptosis has several therapeutic implications. For example, targeting ferroptosis in cancer cells could reverse resistance to immune checkpoint inhibitors. The authors also describe the crucial roles of interferon-γ signaling pathway, which is at the crossroads of ICI therapy and ferroptosis, in modulating the therapeutic outcome.

In conclusion, advances in molecular and cellular technologies have enhanced our understanding of the mechanisms underlying non-apoptotic types of cell death, including pyroptosis and ferroptosis, as well as their role in cancer development and progression. There is also mounting evidence supporting the ability of non-apoptotic cell death pathways to predict patient survival in multiple cancers. Future preclinical and clinical studies are warranted to confirm the prognostic and therapeutic roles of pyroptosis and ferroptosis.

## References

[B1] Nirmala G. J. Lopus M. (2020). Cell death mechanisms in eukaryotes Cell Biol. Toxicol. 36, 145–164. 10.1007/s10565-019-09496-2 31820165

